# How much can farmers pay for weeding robots? A Monte Carlo simulation study

**DOI:** 10.1007/s11119-023-10015-x

**Published:** 2023-04-05

**Authors:** Linmei Shang, Christoph Pahmeyer, Thomas Heckelei, Sebastian Rasch, Hugo Storm

**Affiliations:** 1 Institute for Food and Resource Economics (ILR), University of Bonn, Nußallee 21, 53115 Bonn, Germany; 2 Thünen Institute of Farm Economics, Bundesallee 63, 38116 Braunschweig, Germany

**Keywords:** Weeding robot, Labour, Technology adoption, Supervision, Investment

## Abstract

This paper investigates the Maximum Acquisition Values (MAVs) of weeding robots and their determinants in both organic and conventional sugar beet farming in Germany. The MAV is defined in this paper as the price of the weeding robot that renders the same net profit as the current weeding methods. For our analysis, a Monte Carlo simulation approach is used, combined with empirical data and data collected from weeding robot companies. The results show that the MAVs of mechanical weeding robots for organic farming are substantially higher than that of spot spraying robots for conventional farming. Technology attributes are more influential than labour cost in determining the MAVs of weeding robots: in organic farming, technology attributes such as area capacity and weeding efficiency impact the MAVs of mechanical weeding robots the most; in conventional farming, supervision intensity and the robot’s ability to save herbicides are the most influential factors. The wage rate of unskilled labour, relevant for manual weeding, plays a more important role in determining the MAVs than that of skilled labour, relevant for supervision of the robot. This implies that a shortage of seasonal workers and hence increases in the wage of low-skilled labour could be important drivers of the adoption of mechanical weeding robots. Plot characteristics such as plot size and mechanisation level only have limited impacts on the MAVs.

**Supplementary Information:**

The online version contains supplementary material available at 10.1007/s11119-023-10015-x.

## Introduction

Weed control is a key activity for both organic and conventional farming systems. In organic farming, manual weeding is labour-intensive and increasingly expensive in the European Union due to the shortage of workforce in the agricultural sector (Williams & Horodnic, 2018), which is amplified by the Covid-19 pandemic and the recent war in Ukraine (Bochtis et al., 2020; Dahm, 2022). In conventional farming, chemical weeding methods are effective, but they are usually costly, can create herbicide resistance problems and cause adverse environmental impacts. Thus, the Farm to Fork Strategy of the European Green Deal sets a goal of reducing chemical pesticide use by 50% by 2030 (European Commission, 2022; Montanarella and Panagos, 2021). In addition, farmers face regulatory uncertainties about the future availability of herbicides (see e.g. Stokstad, 2017). Cost-effective and environmentally friendly weeding methods are urgently needed to ensure food security and the sustainability of agriculture in the context of a growing world population (MacLaren et al., 2020).

Autonomous weeding robots have great potential to overcome the challenge of agricultural labour shortage and reduce the negative environmental impacts of agricultural production (Khanna et al., 2022; Lowenberg-DeBoer et al., 2021b; Gallardo & Sauer, 2018). Combining the recent advances in information and communications technology, robotics and artificial intelligence, autonomous weeding robots can distinguish weeds from crops and precisely treat the targeted weeds at the individual plant level (Bawden et al., 2017). Currently, there are many types of weeding robots that are commercialised or in development such as GPS-based mechanical weeding robots (e.g. FD20 of FarmDroid, 2022), vision-based mechanical weeding robots (e.g. Dino of Naïo Technologies, 2022), vision-based selective spot spraying robots (e.g. AVO of EcoRobotix, 2022), and vision-based thermal weed control with laser (e.g. LaserWeeder of Carbon Robotics, 2022).

Despite the rapid advancement in the engineering of agricultural robotics, the economic analysis of agricultural robots has lagged due to their limited adoption and data availability from farm trials (Lowenberg-DeBoer et al., 2020; Spykman et al., 2021). In the review of Lowenberg-DeBoer et al. (2020), only 18 studies that include economic analyses of agricultural automation and robotics are identified. However, economic analyses are highly relevant for farmers’ adoption decisions, technology providers’ machine design (Shockley et al., 2019), and policymakers’ strategies to promote adoption and tackle the uncertainties in the labour market. Therefore, this paper contributes in this regard by conducting a cost-based investment analysis of weeding robots.

The aim of this paper is to investigate the Maximum Acquisition Values (MAV) (Shockley et al., 2019; Sørensen et al., 2005) of weeding robots and their determinants in both organic and conventional sugar beet farming in Germany. Following Shockley et al. (2019) and Sørensen et al. (2005), the MAV of a weeding robot is defined here as the price of the robot that renders the same net profit as the current weeding methods. Specifically, this paper will (1) evaluate the MAVs of weeding robots in both organic and conventional sugar beet farming in Germany; (2) compare the importance of technology attributes and labour cost in determining the MAVs of weeding robots; and (3) examine the impact of plot characteristics on the MAVs of weeding robots. Accordingly, this paper employs a Monte Carlo simulation based on farm planning data extracted from the KTBL (2020) (In German: Kuratorium für Technik und Bauwesen in der Landwirtschaft e.V.; In English: the Association for Technology and Structures in Agriculture) database. To define the ranges of the robot characteristics for the Monte Carlo simulation, relevant information about the characteristics of currently available robots was collected through personal interviews with leading weeding robot companies, information on their homepages, and existing literature. For the sake of simplicity, this paper focuses on sugar beets since there are many weeding robots already available that support the cultivation of sugar beets (Ducksize, 2022).

This paper is organised as follows. It first reviews the economic studies of agricultural robots, especially weeding robots, in the existing literature. Then, the KTBL dataset and the method for calculating the MAVs are introduced. Afterwards, the results are analysed and discussed. The last section concludes the paper and points out its limitations and the directions for future research.

## Literature review

In this section, studies about mechanical weeding and spot spraying robots were firstly summarised, then those on whole-farm autonomous machinery. Some studies that only indirectly investigate the economics of agricultural robots but provide important insights are also reviewed.

One of the earliest economic studies of mechanical weeding robots was conducted by Sørensen et al. (2005). Their intra-row mechanical weeding robot was based on a small autonomous vehicle with vision systems and active tools for weed removal. Their result showed that mechanical weeding robots could reduce the labour use by 85% in organic sugar beet farming and by 60% for organic carrot production in case of 100% weeding efficiency (i.e. the percentage of weed removed). With a 75% weeding efficiency, the labour cost could be reduced by around 50%. They also estimated the MAV of a weeding robot: A farmer could pay up to €110,000 for the weeding robot in case of high weeding intensity and high utilisation level of the robot (300 operation hours per year). With a low weeding intensity and low utilisation level (180 operation hours per year), the MAV was less than €40,000. Pérez-Ruíz et al. (2014) evaluated the labour-saving effect of an intra-row mechanical weeding co-robot on an experimental tomato plot at the University of California. In the cooperation of the co-robot and a human, the human provided visual crop detection capability and manually located the hoes in between row crops, while the co-robot took on the drudgery of repetitive hoe movement. The result showed that using the co-robotic system replaced nearly 60% of hand hoeing labour for intra-row weed control.

Turning to spot spraying robots, Pedersen et al. (2006) compared robotic weeding based on a micro spraying system with a conventional sprayer for sugar beet farming in Denmark. This system could weed 0.4 ha/h, and it was assumed to save herbicide use by 90%. Their economic feasibility assessment showed that robotic weeding was more profitable than conventional systems: The robotic system could reduce operating costs by up to 24%. They also estimated an initial cost of nearly €65,000 for such a weeding robot. Pedersen et al. (2008) extended the study and estimated the costs of a similar robotic weeding system for sugar beet farming in Denmark, the US, the UK and Greece. These countries differ in farm size and labour cost, as well as technical parameters of the robotic weeding system. The results indicated that the robotic weeder had a cost advantage in all study regions except Greece, where the wage rate of unskilled labour was relatively lower than in the other three countries and the total treated area was also smaller.

There are also studies on the economics of autonomous machinery for the whole farming system. Shockley et al. (2019) used whole-farm mixed-integer programming considering the entire farming system to compare the net returns of using conventional and autonomous machinery (including tractor, planter, sprayer, and fertiliser applicator), guided by intelligent controls, for corn and soybean production in Kentucky, USA. They investigated the economic feasibility and break-even investment price of intelligent controls (not including the machinery). For an 850 ha grain farm, the break-even investment price ranged from around $26,000 up to $160,000, depending on the degree of input reduction and yield increasing effect. Their sensitivity analysis on farm size showed that without considering input saving or yield increasing effect, farm size only had a limited impact on the break-even investment price. However, farm size impacted the break-even investment price dramatically when input saving and yield-increasing effects were considered. The study is extended by Shockley et al. (2021). They examined the farm-level implications of on-site supervisory regulations and a speed restriction. These regulations reduced the profitability of autonomous machinery, and in some scenarios, autonomous machines were no longer an economically viable alternative to conventional machinery.

Lowenberg-DeBoer et al. (2021a) went beyond the economic analysis of Shockley et al. (2019) showing it is technically possible to use Global Navigation Satellite Systems and drone autopilot software to retrofit conventional farm equipment to autonomous operation. They used data from the Hands Free Hectare (HFH) project on a grain-oilseed farm in the UK to estimate the whole farm profitability of an autonomous cropping system. The study showed that arable crop production with autonomous equipment was economically feasible. Although autonomous farms had no substantial improvement in gross margins, they had notably higher returns to operator labour, indicating autonomous farming is more beneficial for production systems that require more labour and field operations. The study suggests that using smaller equipment more intensively can decrease equipment investment costs. This also hints at the potential of small robots in utilising small and irregularly shaped farming plots. Lowenberg-DeBoer et al. (2021b) investigated the impact of supervision time of autonomous equipment and farm size on the costs of wheat production in the UK based on the HFH project. The results showed that for a farm of 66 ha, when a 100% supervision time was required, using autonomous equipment had no cost advantage compared to using conventional farming equipment. When more supervision time was required, smaller farms tended to benefit less from autonomous equipment than bigger farms.

Studies that do not directly investigate the profitability of agricultural robots nevertheless provide some important insights. De Witte (2019) calculated the operating costs of large and small machine combinations for grain harvesting and tillage using mainly farm planning data. The study found small machinery for tillage was 7% cheaper than using large machinery if labour costs were not considered, but small machinery got more expensive than the latter when considering labour costs. For harvesting, using large machinery had an economic advantage independent of including labour costs or not. Thus, it is reasoned that small autonomous machines can become cost-competitive for less capital-intensive processes like tillage and seeding. Interviews with AgTech startups conducted by Rübcke von Veltheim et al. (2020) reveal the expectation that field crop robots would first be implemented in specialty crops and organic farming as the economic case for conventional farming is not yet strong enough. They also predicted that farms with larger fields would adopt field crop robots sooner than farms with small fields, irrespective of total acreage, due to logistic costs. Rübcke von Veltheim et al. (2022) further investigated the behavioural intention of German farmers with respect to their future adoption of autonomous field robots. It is found that farmers’ expected performance and trust in technology had a significant positive impact on their intention to adopt autonomous field robots. They suggested that policymakers should create a stable legal situation for autonomous systems to promote the adoption of field robots. Spykman et al. (2021) investigated farmers’ attitudes towards field crop robots in Bavaria, Germany. The study showed larger farms focus more on the economic advantages of robots and prefer large autonomous tractors. In contrast, small-scale or organic farms consider the environmental impacts of robots relatively more important and favour small robots. Organic farming also positively correlates with the intent to invest in field robots. To the authors’ knowledge, quantitative economic analyses explaining these attitudinal results do not yet exist in the current literature.

Based on the literature review above, the following research gaps are identified: (1) no studies have compared the MAVs of weeding robots in organic farming with conventional farming; (2) no studies have compared the importance of different technology attributes, labour cost, and plot characteristics in determining the MAVs of weeding robots; and (3) no studies so far have investigated the economic implications of weeding robot for German sugar beet farms. Given that filling those gaps will provide relevant information for business strategies and the design of policy measures, this paper aims to investigate the MAVs of weeding robots and their determinants in German sugar beet farming of both conventional and organic farming systems.

## Data and method

This section first describes the KTBL dataset, the baseline scenario with current weeding methods (KTBL, 2020), and the robot scenario with weeding robots. Afterwards, the calculation of MAVs is presented based on the two scenarios.

### The KTBL database and the baseline scenario

The KTBL database provides detailed farm planning data for various farm branches such as arable, livestock and horticultural production in Germany. This extensive data source mainly serves as a basis for planning calculations and business assessments on German farms, but it is also regularly used for policy assessments, research and education (Heinrichs et al., 2021). For arable farming, the dataset provides information on crops including yields, revenues, and costs of all individual operations (e.g. seeding, weeding, harvesting). For each operation, labour requirements, machinery costs, and the costs of contractor services are provided. It also includes different types of costs such as variable costs (variable labour costs and variable machine costs), fixed costs (fixed labour and fixed machine costs), and direct costs (e.g. fertiliser and herbicide, etc.).

Note that all data on costs and revenues are provided on a *per ha* basis instead of per farm. Costs and revenue per ha differ from plot to plot. The KTBL database differentiates plots by plot size, mechanisation level (indicating power of the tractor’s engine), farm-plot distance, and yield level. Data on sugar beet production in both organic and conventional farming systems were extracted. For simplicity, this paper only varies plot size and mechanisation level and fixes other plot characteristics. Plot size is chosen because the average cost of setting up a robot for a field depends on the plot size assuming the robot only needs to be set up once per field. The mechanisation level represents the existing technology, thus determining the profit level of the plot. Other plot characteristics are fixed: The farm-plot distance is fixed at 2 km (default assumption used in the KTBL database (KTBL, 2022)), and the yield is fixed at a medium level. Plot size is a discrete variable including {1, 2, 5, 10, 20, 40, 80} ha, and the mechanisation level is also discrete including {45, 67, 83, 102, 120, 200, 230} kW. In total, there are 49 combinations (7 plot sizes × 7 mechanisation levels) of different plot characteristics for organic and conventional farming, respectively.

**Fig. 1:**
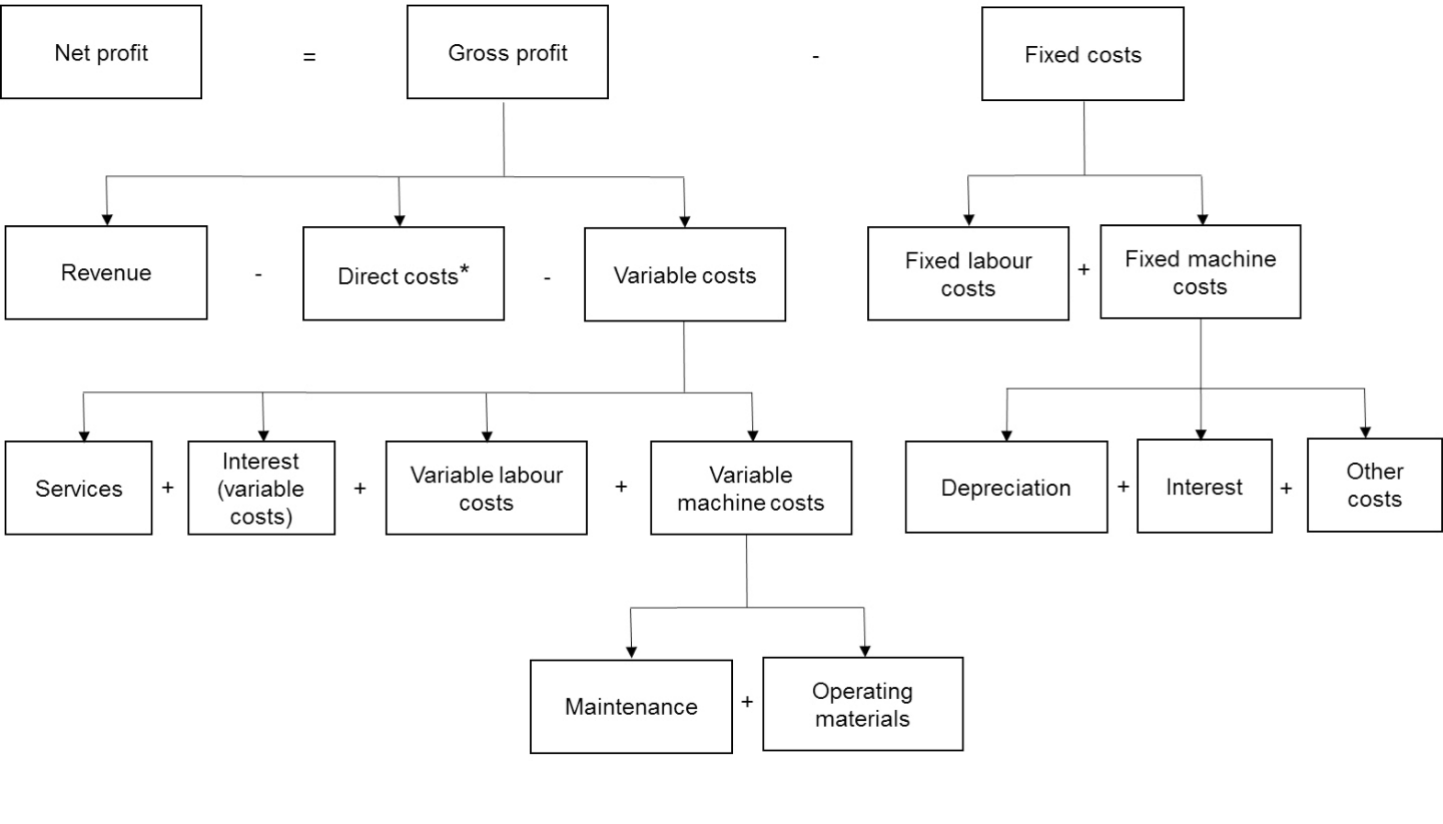
Net profit and cost structure * Note: Direct costs include costs of direct farming inputs, e.g. seeds, fertilisers, herbicides, manure, hail insurance, and lime. Source: based on KTBL (2019, 2020). Graphics programme used: Microsoft PowerPoint.

A plot of size of 10 ha and mechanisation level of 102 kW in organic farming is used to illustrate the dataset: costs of individual operations (Table 1) and revenue and cost structure (Table 2). Table 1 shows the costs of individual operations on a sugar beet plot (not for all farm activities) during one year. The first two columns tell when and what individual operations are implemented (with what type of machinery). The column “Time” provides labour requirements (in hours) of each operation *per ha* during the season. The next five columns present the machinery costs *per ha* of each operation: depreciation, interest (for equity capital and borrowed capital for financing the machinery, see KTBL (2019) for details), other costs, maintenance, operating materials (diesel, gasoline, electricity, etc.). The last column shows the service costs per ha. Note that Table 1 only contains costs of labour, machinery and service but does not include the costs of direct farming inputs (e.g. seeds, manure, lime, hail insurance). For example, the operation “precision sowing” does not show the cost of seeds but the labour requirement and different costs of a sowing machine. Table 2 complements Table 1 by providing direct costs and categorising the costs in Table 1 as variable or fixed costs *per ha*. Therefore, this paper differentiates direct costs and variable costs due to the data structure of KTBL. In addition, Table 2 provides the information of revenue of sugar beet *per ha*. Figure 1 illustrates the relationship among the elements presented in Tables 1 and 2 (KTBL, 2019). For the case of conventional farming, seeTable A.1 and Table A.2 in the appendix.

Specifically, variable machine costs and fixed machine costs are the sum of different elements from Table 1, see Eq. (1) and Eq. (2).

1 $$Variable\,machine\,costs = Sum\,of\,maintenance + Sum\,\,of\,{\rm{operating\, materials}}$$

2 $$Fixed\,machine\,costs = Sum\,of\,depreciation\, + \,Sum\,of\,interest\, + \,Sum\,of\,other\,costs$$

Variable labour costs and fixed labour costs are calculated based on Eqs. (3) and (4). According to the dataset, all field operations in conventional farming are done by permanent farm workers (hired or family labour, calculated as fixed labour cost), requiring no seasonal labour (unskilled labour, calculated as variable labour cost); in organic farming, only manual weeding requires partially unskilled labour. As shown in Table 1 (organic farming), there are multiple weeding steps (i.e. hoeing) from April to June. Normal hoeing is mechanical weeding with a curry-comb carried by a tractor, while hand hoeing stands for manual weeding. According to the assumption of KTBL, mechanical weeding is done by permanent farm workers, while the labour cost of manual weeding consists of 11% fixed labour cost and 89% variable labour cost.

3 $$Variable\,labour\,costs\, = \,Unskilled\,labour\,time \times Wage\,rate\,of\,unskilled\,labour$$

4 $$Fixed\,labour\,costs\, = \,Fixed\,labour\,time\, \times Wage\,rate\,of\,fixed\,labour$$

In the baseline scenario, farmers use the current weeding methods, i.e. manual weeding and mechanical weeding with a tractor in organic farming, and chemical spraying in conventional farming. From Table 2, the net profit (*per ha*) of the baseline scenario (${\pi }_{1}$) is calculated as shown in Eq. (5):

5 $$\eqalign{Net\,profit\, & = \,Gross\,profit-Fixed\,cost\\& = {\rm{ }}Revenue - Total\,direct\,costs - Total\,variable\,costs - Total\,fixed\,costs }$$

**Table 1:** Costs (per ha*) of all individual operations for organic farming and a plot size of 10 ha with mechanisation level of 102 kW

Month	Operations	Time required (h/ha)	Depreciation (€/ha)	Interest (€/ha)	Other costs (€/ha)	Maintenance (€/ha)	Operating materials (€/ha)	Services (€/ha)
OCT	Soil sample	0.02	0.08	0.01	0	0.07	0.01	1.2
OCT	Ploughing with a reversible plough	1.13	16.67	4.85	2.08	20.44	18.99	0
FEB	Harrowing with spring tine harrow	0.32	6.79	2.03	0.94	5.86	4.62	0
FEB	Nmin-sampling, 0–30 cm	0.19	0.64	0.06	0.01	0.53	0.05	2
MAR	Spreading liquid manure, from farm	1.01	17.65	4.33	2.3	14.55	7.34	0
MAR	Harrowing with seedbed combination	0.28	6.73	2.02	0.9	6.06	4.15	0
MAR	Precision sowing	0.44	24	6.48	1.1	11.88	2.69	0
APR	Hoeing, (1) and (2) hoeing	0.49	7.31	1.97	0.69	6.02	3.29	0
MAY	Hoeing, (1) and (2) hoeing	0.49	7.31	1.97	0.69	6.02	3.29	0
MAY	Crop appraisals	0.1	0.08	0.02	0.06	0.03	0.14	0
MAY	Hand hoeing (1. hoeing)	85.43	0.92	0.21	1.33	1.1	2.58	0
MAY	Hoeing, 3. and 4. hoeing	0.41	6.99	1.89	0.64	5.52	2.91	0
JUN	Hand hoeing (at row closing)	77.74	0.86	0.19	1.26	1.04	2.35	0
SEP	Harvesting	1.05	96.66	26.1	5.26	65.14	34.65	0
OCT	Lime fertilisation, wheel loader, mineral fertiliser shovel	0.01	0.13	0.03	0.01	0.08	0.07	0
OCT	Lime fertilisation, mounted spreader	0.03	1.92	0.44	0.2	0.52	0.42	0
OCT	Processing stubbles, flat, sloped (30°)	0.48	8.41	2.47	1.35	8.63	4.97	0
	**Sum**	**169.62**	**203.15**	**55.07**	**18.82**	**153.49**	**92.52**	**3.2**

Source: KTBL (2020)

* Note: The unit of this table is not per year because the costs of each individual operation are calculated by usage of labour and machinery per ha. This is the same for Table 2, where the revenue and costs are calculated per ha instead of per year at farm level.

**Table 2:** Revenue and cost structure (per ha) for organic farming and a plot size of 10 ha with mechanisation level of 102kw

	Detailed Item	Amount	Amount Unit	Price	Price Unit	Total (€/ha)
Revenue	Sugar beet, organic	50	t/ha	105	€/t	5,250
Direct Costs	Seeds, organic	1.23	U/ha	230	€/U	282.9
Direct Costs	Interest (3 month)	91.68	€/ha	0.03	€/€	2.75
Direct Costs	Liquid manure	20	m³/ha	0	€/m³	0
Direct Costs	Calcium carbonate	1	t/ha	40.7	€/t	40.7
Direct Costs	Hail insurance*	5,250	€/ha	8.21	€/1000 €	43.1
Variable Costs	Variable machine costs	/	/	/	/	246.01
Variable Costs	Variable labour costs	145.04	h/ha	13.25	€/h	1,921.78
Variable Costs	Services	/	/	/	/	3.2
Variable Costs	Interest (3 month)	542.75	€/ha	0.03	€/€	16.28
Fixed Costs	Fixes machine costs	/	/	/	/	277.04
Fixed Costs	Fixed labour costs	24.58	h/ha	21	€/h	516.18

Source: KTBL (2020)

* Note: In this table, a farmer pays €8.21 to buy insurance for crops worth €1,000. One hectare of organic sugar beet is estimated to be worth €5,250 as can be seen in the table. Thus, the hail insurance costs 8.21 * 5,250/1,000 = €43.1 per ha.

### Robot scenario

This section describes the assumptions of the robot scenario and the simulated variables in this scenario.

#### Assumptions

1. Two types of weeding robots are used for two farming systems.

It is assumed in this paper that a mechanical weeding robot will be used for organic farming, and a spot spraying robot for conventional farming. The differentiation has been established since labour intensity is a major driver of the costs in organic sugar beet cultivation (see Table 1 and Table 2), whereas in conventional agriculture the cost of herbicides plays a larger role than labour intensity (KTBL, 2020). The exact technical execution of the weed removal is not crucial in this study as long as no chemicals are used in organic farming and conventional farming still uses herbicides to kill weeds.

(2) Operations replaced by weeding robots.

Based on the tables of individual operations from KTBL, it is assumed that weeding robots go through the fields twice per season in both organic and conventional farming. For organic farming (see Table 1), only manual weeding (i.e. hand hoeing) is replaced by a mechanical weeding robot (twice per season, in May and June, respectively) because this paper assumes that normal hoeing with a tractor is efficient enough so that a robot cannot compete with it. If a robot is not able to remove 100% of the weed, the rest will be done by manual weeding (11% fixed labour and 89% variable labour, as assumed by KTBL). In conventional farming, weeding is done by a tractor with a sprayer driven by a permanent farm worker (twice per season, “apply herbicide, sprayer” in March and May, respectively, see Appendix A), thus no unskilled labour is required. This paper assumes that the spot spraying robots are able to kill all weeds in the field, but their ability to save herbicide varies.

(3) Revenue per ha stays the same as in the baseline.

It is assumed that the revenue per ha in the robot scenario is the same as in the baseline for each plot, meaning the quality of crop output and yield stay unchanged. Since the KTBL data only provide the costs and revenue per ha, the costs and revenue in the robot scenario are also calculated per ha. In this way, the MAV is the price of the robot that renders the same net profit per ha as the current weeding methods. Since the KTBL dataset does not provide information on farm size, this paper only focuses on the average profit of each plot, and no farm size is assumed. Thus, our analysis is not at the farm level but focuses on the profit of the production activity.

(4) Robots are operated at full capacity.

The focus on the plot level comes with the assumption that the weeding robots work at full capacity regardless of farm size. This implies either that the farm has the appropriate size, or the remaining capacity can be rented out at rates reflecting the costs. Assuming that a robot works at full capacity may cause an overestimation of MAVs for small farms that do not manage to rent out excess hours. In addition, timing of using a robot is not taken into consideration, meaning all plots can be weeded in time.

(5) Skilled labour for setting up and supervising the robot.

It is assumed that the robot is set up and supervised by skilled labour to ensure safe operations on the field for both organic and conventional farming. Although Shockley et al. (2021) and Lowenberg-DeBoer et al. (2021b) view the required level of supervision time as a regulation, it can also be seen as a technology attribute depending on the levels of autonomy of the robot. For simplicity, this paper includes the required level of supervision time as a technology attribute (see “supervision intensity” in “Variables and the accounting system”).

#### Variables and the accounting system

To calculate the MAVs of weeding robots, variables of technology attributes and their values need to be defined, same for the wage rates of skilled and unskilled labour. Definitions and ranges of value are presented in Table 3. The actual values used to calculate the MAVs are drawn from these ranges in a Monte Carlo simulation. The ranges of the variables come from various sources: personal interviews with leading weeding robot companies, information on their homepages, existing literature, and KTBL database.

Personal interviews with leading weeding robot companies were conducted on the DLG (In German: Deutsche Landwirtschafts-Gesellschaft; In English: German Agricultural Society) field days (14th-16th June 2022, Mannheim, Germany). In total, 7 companies were interviewed, among which 6 companies produce mechanical weeding robots, and 1 company produces spot spraying robots. Those companies already offer commercial robots on the market. During the interviews, the aim was to collect information on the robot characteristics (see variable (1)–(5) below), which is then used to specify their ranges in the Monte Carlo simulation. Experts were asked to estimate these technology attributes according to their knowledge about their products and experience of using them. Variables and their ranges are described in the following.

1. Area capacity.

The area capacity of a weeding robot is measured by the amount of area (in ha) it can weed in its useful life. This information is usually difficult to estimate for technology providers. Thus, the area capacity is approximated based on the lifetime and weeding capacity per year of a robot. The total lifetime of a robot is assumed to be 10 years according to Sørensen et al. (2005), Pedersen (2006 and 2008), and FarmDroid (2022), which is also similar to the average lifetime of hoeing equipment and self-propelled machinery. According to FarmDroid (2022), the mechanical weeding robot FD20 is designed to farm up to 20 ha per season. When assuming weeding twice per year and 10 years of useful life, the area capacity is 400 ha. According to the personal interviews, three other robot companies also estimated a similar capacity for their robots. Although spot spraying robots should have higher area capacity because of their faster speed, due to the lack of data, this paper uses 400 ha as an average level and set a range between 200 to 600 ha for area capacity for both types of robots. This allows us to compare the MAV of the two types of robots assuming they have the same characteristics.

(2) Setup time per plot.

The setup time per plot is defined as the time required for preparing the robot for the actual fieldwork. According to robot companies, the setup of the first time involves settling the GPS station and loading the map, which takes about several hours. But from the second time, each setup per plot only needs from 10 minutes to 2 h depending on the situation. Therefore, the range from 0.16 h to 2 h is chosen for this variable. It is assumed that a robot must only be set up once for a whole plot irrespective of plot size.

(3) Repair and energy costs.

Repair and energy costs are difficult to estimate for technology providers because they do not have enough experience yet. Therefore, this paper uses the KTBL data for a standard tractor (all-wheel drive, manual gearbox, 40 km/h, 102 kW) and attached hoeing machine (3 m, row width 45-50 cm, 6 rows). The combined repair and energy costs for this combination are 28 €/ha. Since the weeding robot can be solar-powered and the maintenance costs might differ among different robots, for the analysis, the range is assumed to be a minimum of half the respective costs (14 €/ha), and a maximum of twice the costs of the standard tractor (56 €/ha).

(4) Weeding efficiency.

The weeding efficiency of the two types of weeding robots is defined differently. For a mechanical weeding robot, weeding efficiency measures the percentage of weeds that can be autonomously removed by the robot, whereas for a spot spraying robot, it measures the percentage of herbicide that can be saved (compared with the baseline). According to the information collected from robot companies, the efficiency of a mechanical weeding robot ranges from 70–99%, which is similar to Bawden et al. (2017) and Kunz et al. (2015). The spot spraying robot can save up to 95% herbicide depending on the weed density (see e.g. EcoRobotix, 2022). The spot spray technology See & Spray of John Deere (2021) can reduce herbicide use by 77% on average. Thus, a minimal weeding efficiency of 50% and a maximum weeding efficiency of 100% are assumed for both types of robots.

(5) Supervision intensity.

Supervision intensity is defined in this paper as a fraction of the field time, which is the same as the level of supervision time in Lowenberg-DeBoer et al. (2021b) and Shockley (2021). This study assumes a field time (i.e. weeding time) of 3.2 h/ha for a mechanical weeding robot based on the information collected from the internet (FarmDorid, 2022; Farmers Weekly, 2021; Naïo Technologies, 2022) and through personal interviews with robot companies. Spot spraying robots are usually faster, for example, the AVO of Ecorobotix needs 1.6 h/ha (Ecorobotix, 2022). Due to the limited number of observations for spot spraying robots, and for the sake of comparability, this paper also uses 3.2 h/ha as the field time for spot spraying robots in this study. As the requirement regarding supervision intensity is uncertain, this paper uses a range from 0 to 100% for this variable.

(6) Wage rate of unskilled labour.

Of relevance is the wage rate of seasonal labour hired to remove weeds for organic farming. This variable is assumed to be irrelevant in conventional farming as weeds are not removed manually in this production system. The wage rate of unskilled labour in the KTBL database is assumed as a minimum (13.25 €/h), and the maximum is set to 21 €/h, which is the wage rate of the permanent farm worker according to KTBL (2020). Wage rates include employer contributions to social security.

(7) Wage rate of skilled labour.

This paper considers the wage rate of the skilled labour hired to set up and supervise the robot. The minimum is assumed to be the same as the wage rate of a permanent farm worker in KTBL database (21 €/h), and the maximum is assumed to be twice as much as the minimum (42 €/h). Wage rates include employer contributions to social security.

**Table 3:** Definitions and ranges of variables in the Monte Carlo simulation

Variable	Definition	Range (unit)
Area capacity	The amount of area the robot can weed in its useful life	200–600 (ha)
Setup time per plot	Time required to set up the robot per plot	0.16-2 (h/plot)
Repair and energy costs	Repair and energy costs of the robot for weeding one ha	14–56 (€/ha)
Weeding efficiency	Percentage of weeds removed by the robot (organic farming); Percentage of herbicide saved by the robot (conventional farming)	50-100%
Supervision intensity	Percentage of field time required to supervise the robot	0-100%
Wage rate of unskilled labour	Wage rate of seasonal labour	13.25-21 (€/h)
Wage rate of skilled labour	Wage rate of the personal who sets up and supervises the robot	21–42 (€/h)

Note: All variables will be drawn from uniform distribution.

#### Costs in the robot scenario

The costs of operations that are not replaced by the robot will stay the same as for the baseline. Depending on the farming system and the weeding efficiency of the robot, weeding steps might be partially or completely replaced by the robot. For organic farming, if the weeding efficiency is below 100%, the manual weeding steps can only be partially replaced because the rest of the weeds that are overlooked by the robot must be removed by humans (both fixed and variable labour costs are involved). This causes additional labour and machine costs. The additional costs are fractions of the original costs of baseline depending on the weeding efficiency. For conventional farming, it is assumed that weeding steps are completely replaced by the weeding robots, which means the robot can always achieve the required weeding efficiency. The weeding efficiency only determines how much herbicide, thus the direct costs, can be saved by the spot spraying robot.

In both organic and conventional sugar beet farming, there are weeding steps in the baseline that will be replaced by robotic weeding in our simulation. Following the structure of Table 1, the costs of *one* robotic weeding step *per ha* are shown below.

1. Time (h/ha): it is the labour requirement of robotic weeding per ha. In this study, it is the sum of the setup time and supervision time per ha as shown in Eq. (6), where field time per ha is fixed at 3.2 h/ha as shown above. Since both setup and supervision are assumed to be conducted by skilled labour paid on an hourly basis, these costs will be counted as variable labour costs.

6 $$Time = \frac{{{{Setup\, time\, per\, plot}}}}{{{{Plot\, size}}}} + Supervision\,intensity \times Field\,time\,per\,ha$$

(2) Depreciation (€/ha): the depreciation cost per ha is the MAV of the robot divided by the area capacity because this paper assumes that the robot depreciates linearly by usage. Thus, depreciation in this paper is not calculated per year but per ha. The MAV will be an unknown variable that must be solved in our simulation.

7 $$Depreciation = \frac{{MAV}}{{{{Area\,capacity}}}}$$

(3) Interest and other costs (€/ha): they are assumed to be fractions of the depreciation due to the limited data. Based on the KTBL data of the current machinery, this paper assumes that interest equals to 30% of the depreciation, while other costs equal to 10% of the depreciation.

8 $$Interest = Depreciation \times 0.3$$

9 $$Other\, costs = Depreciation \times 0.1$$

(4) Maintenance and operating materials (€/ha): these two items are merged into “Repair and energy costs”.

10 $$Maintenance + Operating\,materials = Repair\,and\,energy\,costs$$

(5) Services (€/ha): no costs of services are calculated because the costs of hiring skilled labour to set up and supervise the robot are counted as variable labour costs.

As long as the values of the parameters are determined, the costs of each operation in the robot scenario are also determined using Eq. (6) to Eq. (10), assuming the costs of other steps stay the same as in the baseline. Then, different cost categories in the robot scenario can also be calculated. Variable machine costs and fixed machine costs still follow Eq. (1) and Eq. (2). Variable labour costs are calculated based on Eq. (11) since skilled labour is introduced. Direct costs and fixed labour costs are proportions of the corresponding costs in baseline, depending on the weeding efficiency.

11 $$\eqalign{Variable\, labour\, costs & = Skilled\, labour\, time \times Wage\, rate\, of\, skilled\, labour \cr & + Unskilled\, labour\, time \times Wage\, rate\, of\, unskilled\, labour}$$

At the end, the net profit per ha of the robot scenario (${\pi }_{2}$) can also be calculated using Eq. (5).

**Fig. 2:**
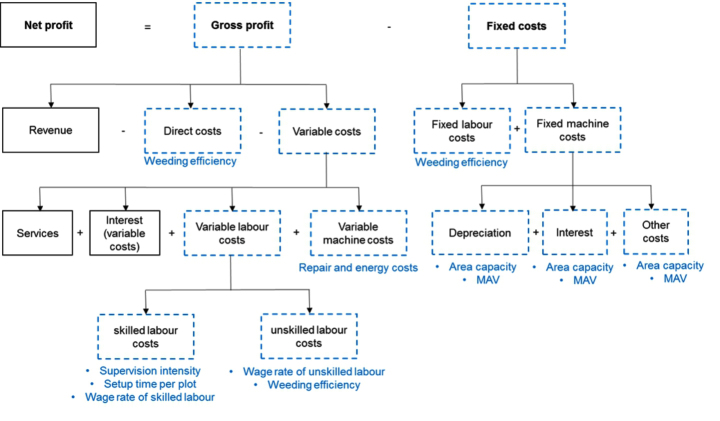
How each variable influences the net profit per ha Note: MAV is not drawn from the Monte Carlo simulation but will be derived from the system. Source: based on KTBL (2019, 2020). Graphics programme used: Microsoft PowerPoint.

Figure 2 illustrates how each variable influences the net profit of using robotic weeding. The types of costs that will change in the robot scenario are marked in dotted boxes, under which the variables that influence them are noted.

### Data generation and calculation of MAVs

**Fig. 3:**
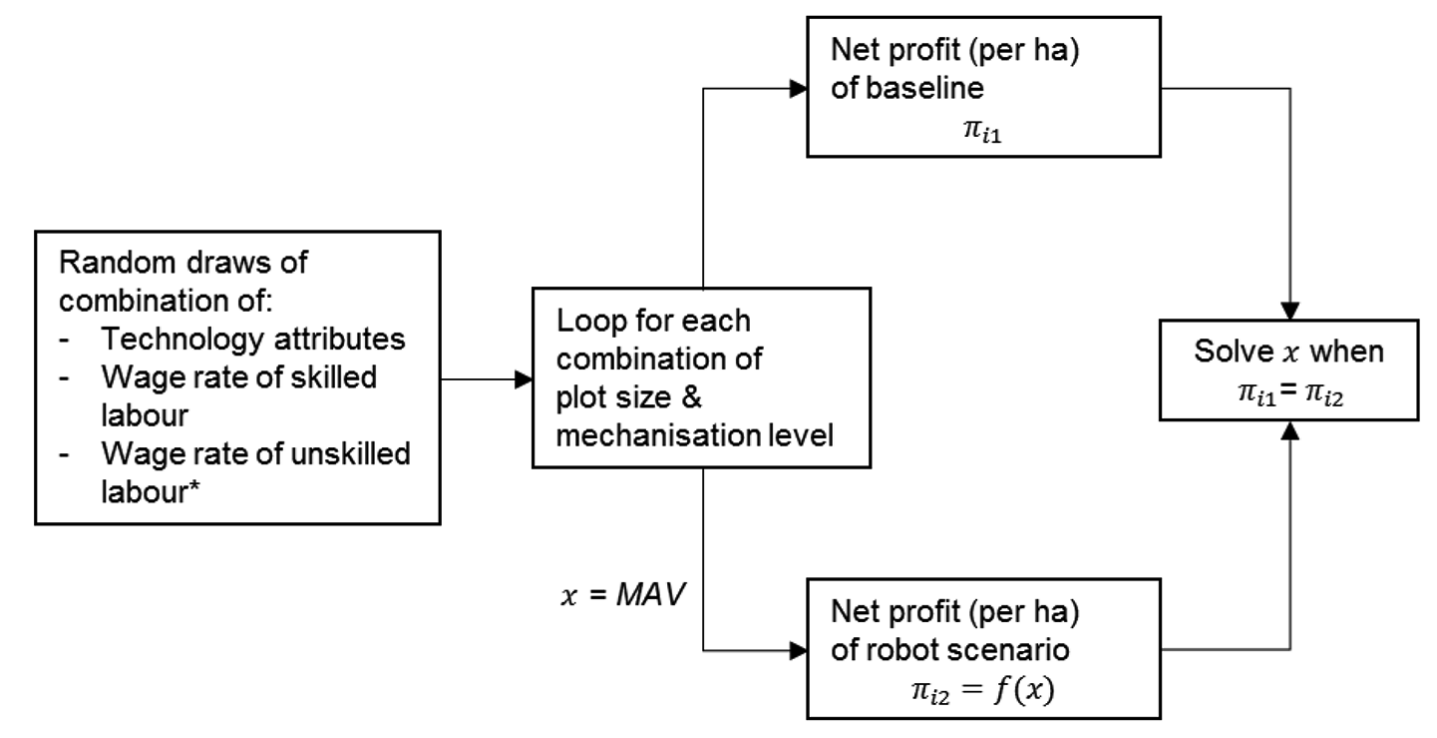
Data generation and the calculation of the MAV of one random draw * Note: only for organic farming. Source: authors’ own figure. Graphics programme used: Microsoft PowerPoint.

Figure 3 depicts the data generation process and the derivation of MAV of one random draw. The data generation processes of organic farming and conventional farming are separated. Here, the process for organic farming is described as an example. First, distributed random outcomes of all variables that matter (7 for organic farming, 6 for conventional farming) are uniformly drawn from the ranges specified above. Then, for each combination of plot size and mechanisation level *i* (49 combinations for both organic and conventional farming), the net profits per ha of baseline and robot scenario are calculated given the randomly drawn values. For organic farming, the net profit per ha of baseline (${\pi }_{i1}$) is calculated given the randomly drawn wage rate of unskilled labour (this step is unnecessary for conventional farming because unskilled labour is not used there). The net profit per ha of the robot scenario (${\pi }_{i2}$) is a function of technology attributes, wage rates of skilled and unskilled labour, and an unknown MAV. The *fsolver* of the *scipy* library (Virtanen et al., 2020) finds the *MAV* that equalises net profits (i.e. ${\pi }_{i1}$= ${\pi }_{i2}$) and implicitly determines the MAV.

The same process is repeated for each draw. A large number of draws is needed to obtain a fairly accurate representation of the results MAV distribution given the multi-dimensional parameter space. In this way, a large dataset consisting of the MAVs and the variables is generated. For organic farming, 32,000 data points were drawn for each combination of plot size and mechanisation level. In the end, 1,568,000 possible data points for all organic farms (32,000 $\times 49$) are generated. For conventional farming, 12,000 data points were drawn for each combination, resulting in 588,000 data points.

## Results and discussion

### Range of MAVs in two farming systems

**Fig. 4:**
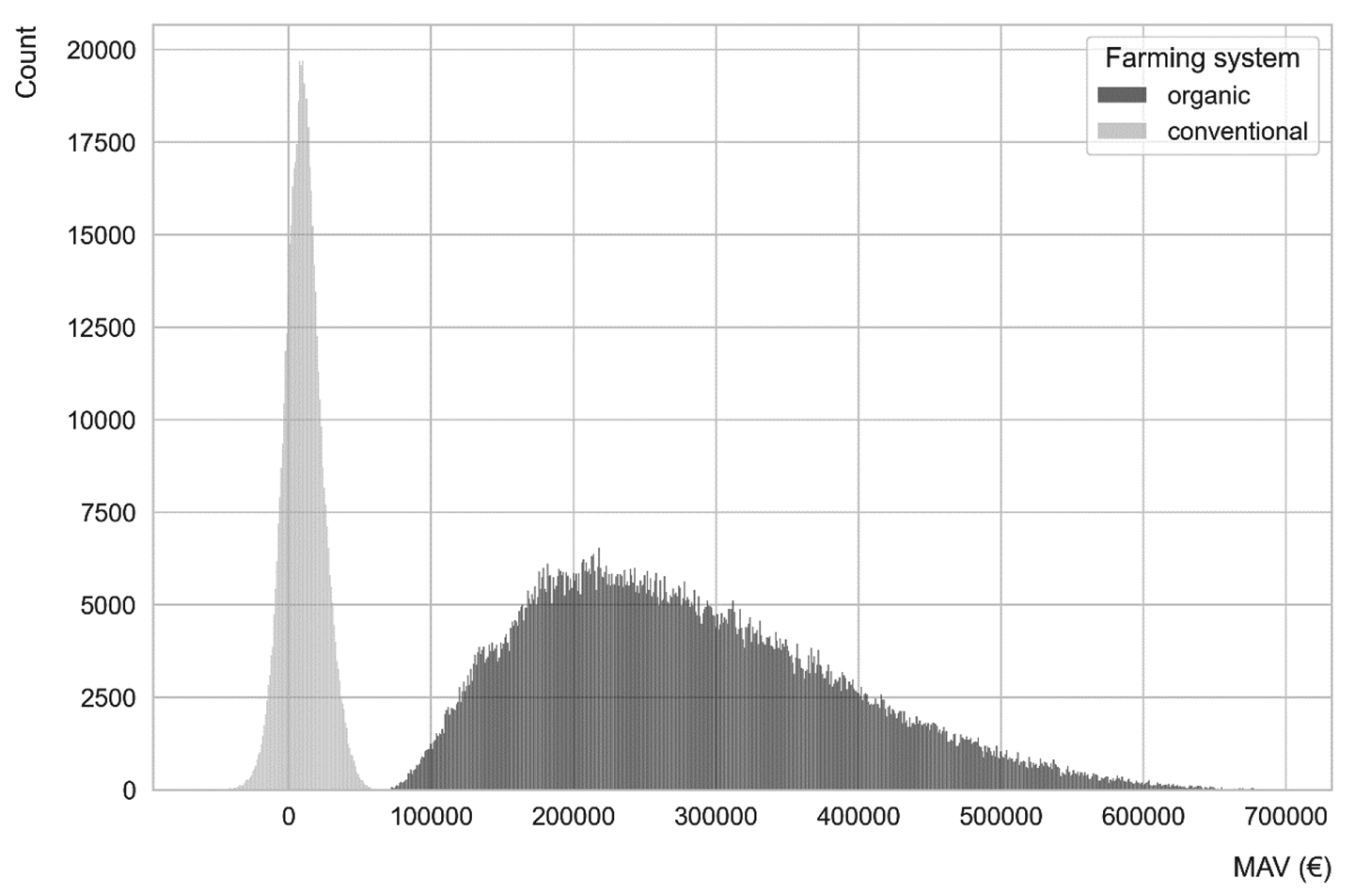
Histograms of the MAVs of weeding robots in organic and conventional sugar beet farming systems Note: Organic farming system contains 1,568,000 data points, and conventional farming contains 588,000 data points. Source: simulation results. Graphics programme used: Python.

Figure 4 shows the distributions of the MAVs in organic and conventional sugar beet farming systems in Germany. MAVs in the organic farming system are distinctly higher than in conventional farming. The MAVs of mechanical weeding robots in organic farming range from €62,564 to €694,073 with a mean of €279,884. In contrast, the MAVs of spot spraying robots in conventional farming have a maximum of €63,364 and a mean of €10,362. Around 21% of the data points have negative MAVs in conventional farming, which means under certain conditions, a compensation to farmers for using the robot would actually be required to keep the same profitability as in the baseline. The partially negative MAVs for spot spraying robots in conventional farming also imply that a good technological performance (e.g. higher area capacity, higher weeding efficiency, less repair and energy costs, etc.) is needed for creating positive MAVs.

The higher variability of the MAVs of weeding robots in organic farming compared with conventional farming largely reflects a higher sensitivity to the changes in the randomly drawn variables given that the ranges and distributions of those are the same for the two types of robots.

The implication of our result is consistent with the findings by Rübcke von Veltheim et al. (2020) and Spykman et al. (2021). The higher MAVs of weeding robots in organic farming mean that organic farms (especially for high-value crops) can pay much more for weeding robots to obtain the current profit level, thus having a stronger economic incentive to adopt autonomous weeding robots than conventional farms. Besides, the availability of weeding robots (and generally agricultural robots) might change the conversion consideration of conventional farms, for whom the high labour requirement has been an obstacle to convert to organic farming (Olabisi et al., 2015).

According to the personal interviews, the price of a mechanical weeding robot ranges from €75,000 to €240,000 (6 robots, average price around €130,000), while the price of a spot spraying robot ranges from €80,000 to €100,000 (2 robots). Comparing the result of the Monte Carlo simulation above, one can see that the profitability margin of organic farms would allow them to pay even more than the current price of mechanical weeding robots to maintain the current profit level. On the contrary, the price of spot spraying robots is much higher than the MAVs from the simulation. It is possible that the technology performance of spot spraying robots is underestimated because of limited observations and limited experience of technology providers so far. For example, the range of area capacity of spot spraying robots is probably underestimated since they have the same range of area capacity with mechanical weeding robots in this simulation.

### Importance of different factors

To compare the importance of different factors, Table 4 is constructed to show how the average MAV changes across each quarter of the simulated range of each factor (averaging across the outcome for all simulations, i.e. averaging across the other variables). For example, for the variable “area capacity” (ranging from 200 to 600 ha), the range is split into four quarters to calculate the average MAV of all data points. €175,650 is the average MAV of those data points whose area capacity is between 200 ha and 300 ha in organic sugar beet farming. Besides, for each variable, Table 4 also presents the change of MAV from Q1 to Q4 ($\varDelta$MAV), which measures the importance of the variable in determining the MAV of a weeding robot considering the assumed scale of the variable.

According to this measure, the most important factor in determining the MAVs of weeding robots in organic farming is the area capacity of a weeding robot. When the area capacity increases from a low level (Q1: 200–300 ha) to a high level (Q4: 500–600 ha), the average MAV increases by €208,741. Weeding efficiency (i.e. the percentage of weeds removed by the mechanical weeding robot in organic farming) is the second most important factor. A robot that can remove 87.5-100% (Q4) of the weeds can attract farmers to pay €150,118 more than a robot with an efficiency between 50% and 62.5% (Q1). In terms of labour cost, the wage rate of unskilled labour has a larger impact than the wage rate of skilled labour on the MAV of a weeding robot in organic farming: the $\varDelta$MAV of the wage of unskilled labour is €87,345, but €-9,345 for the wage rate of skilled labour. This is because changes in the wage rate of unskilled labour influence the production cost much greater than the wage rate change in skilled labour. This finding implies that increasingly more expensive seasonal labour could be one important driver for adopting mechanical weeding robots in organic farming. Supervision intensity is the fourth most important factor among the seven factors in influencing the MAV of a weeding robot. When the supervision intensity increases from Q1 (0-25%) to Q4 (75-100%), the MAV of a mechanical weeding robot would drop by €21,111. The impacts of repair and energy costs and setup time per plot are less influential compared to other factors.

In conventional sugar beet farming, the most influential factor is supervision intensity. As can be seen, when supervision intensity increases from Q1 (0-25%) to Q4 (75-100%), the MAV of a spot spraying robot would drop by €21,934. Both Shockley et al. (2021) and Lowenberg-DeBoer et al. (2021b) found that high supervision intensity can lead to a negative profit level in conventional farming. When only looking at the data points with negative MAVs, the average supervision intensity is 77% (not shown in the table). This result corresponds with the study of Lowenberg-DeBoer et al. (2021b). They found that for a 66 ha farm, using autonomous equipment had no cost advantage anymore when 100% supervision was required. In our simulation, the second most important factor is weeding efficiency (i.e. the percentage of herbicide saved by the spraying robot in conventional farming). Farmers can pay €17,285 more for a spraying robot that can save herbicide by 87.5-100% (Q4) than a robot that is only able to save 50-62.5% (Q1) of the herbicide use. Repair and energy costs and the wage rate of skilled labour are of similar importance in determining the MAV. In conventional farming, the area capacity of a weeding robot is much less influential than in organic farming because the economic benefit per ha of using a weeding robot is less than that in organic farming. However, when considering the environmental impact of applying less herbicide, with policy incentives, conventional farmers might be willing to switch to robotic weeding methods.

In both farming systems, setup time per plot plays the least important role in determining the MAV of a weeding robot because the setup cost is only a minor part of the production costs. In general, a longer setup time per plot will decrease the MAV of a weeding robot. However, the differences in MAVs seem to be quite small, in some cases (e.g. in organic farming from Q3 to Q4) even smaller than the sampling noise.

Given that the first two most important factors in organic and conventional farming are technology attributes, the advancement of technology seems to be more relevant for changes in MAVs than changes in the labour market. However, this needs to be cautiously interpreted because the selected technology and labour market characteristics may not accurately reflect current or future changes.

**Table 4:** Average MAV of each quarter and the change of MAV from Q1 to Q4

*Organic sugar beet farming* (Mechanical weeding robots)	Average MAV (€) (Q1)	Average MAV (€) (Q2)	Average MAV (€) (Q3)	Average MAV (€) (Q4)	$\varDelta$MAV (€) (Q4-Q1)
Area capacity (200–600 ha)	175,650	245,224	314,760	384,391	208,741
Setup time per plot (0.16-2 h/plot)	281,580	280,301	278,674	278,956	-2,624
Repair and energy costs (14–56 €/ha)	284,029	280,537	278,316	276,700	-7,330
Weeding efficiency (50-100%)	204,185	255,289	307,269	354,303	150,118
Supervision intensity (0-100%)	290,236	283,126	277,182	269,125	-21,111
Wage rate of skilled labour (21–42 €/h)	285,200	281,143	277,383	275,855	-9,345
Wage rate of unskilled labour (13.25-21 €/h)	234,391	266,080	296,884	321,736	87,345
***Conventional sugar beet farming*** (Spot spraying robots)	**Average MAV (€)** **(Q1)**	**Average MAV (€)** **(Q2)**	**Average MAV (€)** **(Q3)**	**Average MAV (€)** **(Q4)**	$\varDelta$ **MAV (€)** **(Q4-Q1)**
Area capacity (200–600 ha)	6,473	9,324	11,476	14,107	7,634
Setup time per plot (0.16-2 h/plot)	11,984	10,716	9,953	8,830	-3,154
Repair energy costs (14–56 €/ha)	15,228	11,922	8,975	5,244	-9,984
Weeding efficiency (50-100%)	1,950	7,430	13,067	19,235	17,285
Supervision intensity (0-100%)	21,170	13,913	6,637	-764	-21,934
Wage rate of skilled labour (21–42 €/h)	14,436	11,833	8,987	6,153	-8,283

Source: simulation results

### Impact of plot characteristics

**Fig. 5:**
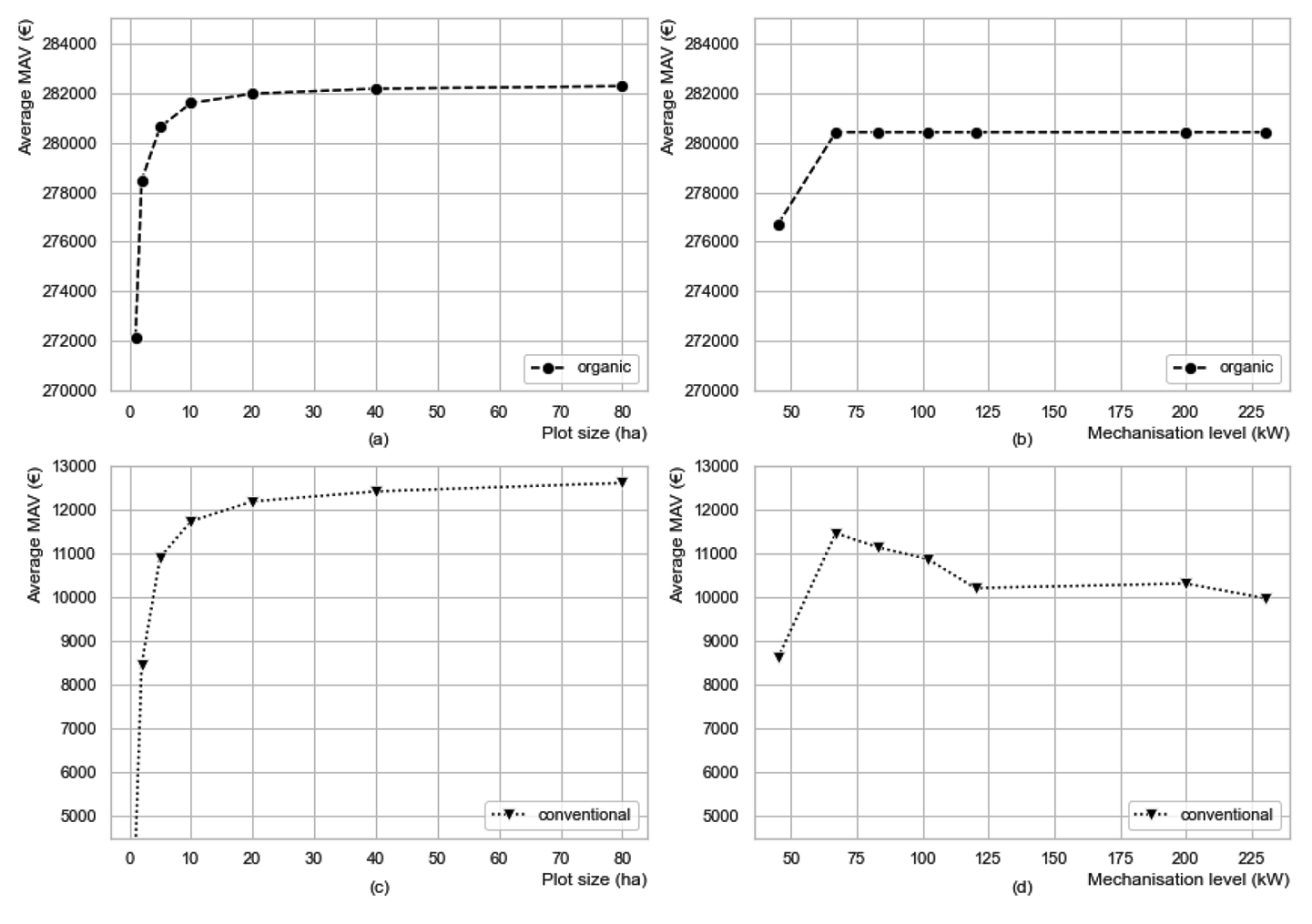
Average MAVs of weeding robots of each plot size and mechanisation level in both farming systems Source: simulation results. Graphics programme used: Python.

Figure 5(a) and Figure 5(b) show the average MAV of each plot size and mechanisation level in organic sugar beet farming across all simulation data points. When the plot size increases from 1 ha to 10 ha, the average MAV increases by €9,451, which is only 3.4% of the mean MAV (€279,884) of a weeding robot in organic farming. From 10 ha to 80 ha, there is only a minor increase in MAV. Regarding the mechanisation level, the MAV of a robot that operates on a plot with a mechanisation level of 67 kW is €3,712 higher than that of a plot with a mechanisation level 45 kW. But the average MAV of farms with a mechanisation level beyond 67 kW does not change. It is because KTBL assumes that beyond 67 kW, the production costs (specifically machine costs and labour costs) do not change for organic farming even though the mechanisation level increases.

Figure 5(c) and Figure 5(d) show the average MAV of each plot size and mechanisation level in conventional sugar beet farming. When the plot size increases from 1 ha to 10 ha, the average MAV increases by €7,468, which is 72% of the mean MAV (€10,362) of a weeding robot in conventional farming. From 10 ha to 80 ha, the average MAV goes up only slightly. It can be observed that the impact of plot size in conventional farming is bigger than that in organic farming. This indicates that a sprayer can work more efficiently on larger fields due to less turning time compared to smaller fields. However, in organic farming, the time requirement of manual weeding (per ha) stays relatively stable as the plot size increases. In terms of mechanisation level, the average MAV is the highest when the mechanisation level is 67 kW. From 67 kW to 120 kW, the average MAV decreases because the average spraying cost goes down as the mechanisation level increases. However, with a mechanisation level of 120 kW, KTBL assumes there is another person driving a water tank when spraying. This paper will not dig into the assumptions of KTBL but focuses on the overall implication of the results of the two farming systems: when the mechanisation level is above 40 kW, a higher mechanisation level reduces the MAVs of spot spraying robots but has no influence on the MAVs of mechanical weeding robots based on the assumptions of KTBL data.

Comparing the changes in MAVs caused by plot characteristics with the $\varDelta$MAVs (Q4 - Q1) caused by technology attributes and labour cost, it can be seen that plot characteristics have only limited importance in determining MAVs of weeding robots in both farming systems.

## Conclusion

This paper investigates the MAVs of weeding robots and the importance of factors from different categories (including technology attributes, labour cost, and plot characteristics) in determining MAVs of weeding robots in German sugar beet farming. It uses a Monte Carlo simulation approach combined with empirical data of KTBL and assumptions about different robotic characteristics based on the information collected from weeding robot companies. The MAV is defined as the break-even investment price that renders the same net profit level as using the current weeding methods.

Under the assumption that mechanical weeding robots replace manual weeding in organic farming, and spot spraying robots replace untargeted herbicide spraying in conventional farming, and considering plausible ranges for the robot characteristics, the results show that the MAVs of mechanical weeding robots in organic farming range from €62,564 to €694,073 with a mean of €279,884. In contrast, the MAVs of spot spraying robots in conventional farming have a maximum of €63,364 and a mean of €10,362. The huge difference in MAVs between organic and conventional farming systems indicates that the economic benefit of mechanical weeding robots for organic farming surpasses that of spot spraying robots for conventional farming, and organic farms are able to pay considerably more for a weeding robot than conventional farms to maintain the current net profit level. Therefore, the adoption and diffusion of weeding robots might also start among organic farms, which is consistent with the findings from previous qualitative studies. Another implication is that the availability of weeding robots (and generally agricultural robots) might change the conversion decision of conventional farms, for whom the high labour requirement could be an obstacle so far.

This paper also quantifies and compares the importance of factors in determining the MAVs of weeding robots from different categories given the chosen ranges of such factors. Firstly, technology attributes are more influential than labour cost in determining the MAVs of weeding robots. For organic farming, the area capacity of a robot impacts its MAV the most, followed by weeding efficiency (the percentage of weeds that can be removed by the mechanical weeding robot). For conventional farming, supervision intensity is the most influential factor, and weeding efficiency (the percentage of herbicide that can be saved by the spot spraying robot) is the second. Secondly, the wage rate of unskilled labour has a larger impact than the wage rate of skilled labour on the MAV of a weeding robot in organic farming because of the high share of unskilled labour costs in the total production costs. The implication is that the shortage in seasonal labour could be one important driver for adopting mechanical weeding robots in organic farming. Thirdly, supervision intensity is the most influential factor in determining the MAVs of spot spraying robots. Our results indicate that high supervision costs in robotic weeding can cause economic infeasibility in conventional farming. In addition, this paper finds that plot characteristics have limited importance in determining the MAVs of weeding robots, compared to technology attributes and labour cost.

This paper innovates by comparing the importance of factors from different categories (technology attributes, labour cost, and plot characteristics) in determining the MAVs of weeding robots in both organic and conventional farming systems. Our approach allows us to experiment with different performances of weeding robots and changes in the labour market. One of the limitations of this study is that the robot scenario does not consider the changes in crop yield and quality, the alternative use of the farm labour after adopting weeding robots, and the environmental impacts at both farm and regional levels due to the lack of data. Future research can make use of data collected by large-scale on-farm precision experimentations (Bullock et al., 2019) with input use decisions and precision and autonomous farming equipment to capture not only the economic but also environmental impacts. Furthermore, this study assumes that the weeding or spot spraying robot has a single purpose. However, there are also multipurpose agricultural robots on the market. For example, the multipurpose tool carrier ROBOTTI (AGROINTELLI, 2023) can be used for e.g. seeding, weeding, ridging, and spraying. Autonomous tractors (e.g. AgXeed, 2023) might also be the future form of the current tractor. By spreading the cost of the base unit over more management activities, the weeding cost should be lower compared to a single purpose equipment.

### Electronic supplementary material

Below is the link to the electronic supplementary material.

Supplementary Material 1

## Data Availability

The data and code used for this paper can be found in the following Github repository: https://github.com/linmeishang/RobotPaperGit.

## Appendix A

**Table A.1:** Costs (per ha) of all individual operations for conventional farming and a plot size of 10 ha with mechanisation level of 102 kW

Month	Operations	Time required (h/ha)	Depreciation (€/ha)	Interest (€/ha)	Other costs (€/ha)	Maintenance (€/ha)	Operating materials (€/ha)	Services (€/ha)
SEP	Soil sample	0.02	0.08	0.01	0	0.07	0.01	1.2
OCT	Apply mineral fertiliser, fertiliser screw conveyor	0.03	0.08	0.02	0.03	0.02	0.04	0
OCT	Apply mineral fertiliser, mounted fertiliser spreader	0.18	1.83	0.45	0.22	1.4	0.94	0
OCT	Ploughing with a reversible plough	1.13	16.67	4.85	2.08	20.44	18.99	0
FEB	Nmin-sampling, 0–30 cm	0.19	0.64	0.06	0.01	0.53	0.05	2
MAR	Harrowing with seedbed combination	0.28	6.73	2.02	0.9	6.06	4.15	0
MAR	Apply mineral fertiliser, fertiliser screw conveyor	0.02	0.05	0.01	0.02	0.01	0.03	0
MAR	Apply mineral fertiliser, mounted fertiliser spreader	0.15	1.36	0.34	0.17	1.13	0.78	0
MAR	Precision sowing	0.44	24	6.48	1.1	11.88	2.69	0
MAR	Weed rating	0.1	0.12	0.03	0.08	0.05	0.18	0
MAR	Apply herbicide, mounted sprayer	0.18	4.12	0.96	0.29	1.69	0.75	0
MAY	Apply herbicide, mounted sprayer	0.18	4.12	0.96	0.29	1.69	0.75	0
JUL	Crop appraisals	0.1	0.08	0.02	0.06	0.03	0.14	0
AUG	Apply fungicide, mounted sprayer	0.18	4.12	0.96	0.29	1.69	0.75	0
SEP	Harvesting	1.11	101.97	27.53	5.55	68.72	38.25	0
OCT	Lime fertilisation	0.01	0.13	0.03	0.01	0.08	0.07	0
OCT	Lime fertilisation, mounted fertiliser spreader	0.03	1.92	0.44	0.2	0.52	0.42	0
OCT	Processing stubbles, flat, sloped (30°)	0.48	8.41	2.47	1.35	8.63	4.97	0
	**Sum**	**4.33**	**168.02**	**45.17**	**11.3**	**116.01**	**68.99**	**3.2**

Source: KTBL (2020)

**Table A.2:** Revenue and cost structure (per ha) for organic farming and a plot size of 10 ha with mechanisation level of 102 kW

	Detailed Item	Amount	Amount Unit	Price	Price Unit	Total (€/ha)
Revenue	Sugar beet	60	t/ha	35	€/t	2,100
Direct Costs	Seeds	1.23	U/ha	180	€/U	221.4
Direct Costs	Interest (3 month)	229.76	€/ha	0.03	€/€	6.89
Direct Costs	Potash and magnesium	140	kg/ha	0.38	€/kg	53.2
Direct Costs	Calcium ammonium nitrate	400	kg/ha	0.23	€/kg	92
Direct Costs	Calcium carbonate	1	t/ha	40.7	€/t	40.7
Direct Costs	phosphorus/potassium fertiliser	450	kg/ha	0.22	€/kg	99
Direct Costs	Fungicide, intensity level 2	/	/	/	/	75.5
Direct Costs	Herbicide, intensity level 2	/	/	/	/	320
Direct Costs	Hail insurance	2,100	€/ha	8.21	€/1,000 €	17.24
Direct Costs	Water	900	l/ha	0	€/l	0
Variable Costs	Variable machine costs	/	/	/	/	198.6
Variable Costs	Variable labour costs	/	h/ha	13.25	€/h	0
Variable Costs	Services	/	/	/	/	3.2
Variable Costs	Interest (3 month)	50.45	€/ha	0.03	€/€	1.5
Fixed Costs	Fixes machine costs	/	/	/	/	236.72
Fixed Costs	Fixed labour costs	4.81	h/ha	21	€/h	101.01

Source: KTBL (2020)

## References

[CR1] AGROINTELLI (2023). *Robotti*. https://agrointelli.com/robotti/, last accessed on 25/01/2023.

[CR2] AgXeed (2023). *AgBot 5.115T2*. https://www.agxeed.com/our-solutions/agbot-5-115t2/, last accessed on 25/01/2023.

[CR3] Bawden O, Kulk J, Russell R, McCool C, English A, Dayoub F, Lehnert C, Perez T (2017). Robot for weed species plant-specific management. Journal of Field Robotics.

[CR4] Bochtis D, Benos L, Lampridi M, Marinoudi V, Pearson S, Sørensen CG (2020). Agricultural Workforce Crisis in Light of the COVID-19 pandemic. Sustainability.

[CR5] Bullock DS, Boerngen M, Tao H, Maxwell B, Luck JD, Shiratsuchi L, Puntel L, Martin NF (2019). The Data-Intensive Farm Management Project: Changing Agronomic Research through On‐Farm Precision Experimentation. Agronomy Journal.

[CR6] Carbon Robotics (2022). *Autonomous Laserweeder Demo Unit*. https://carbonrobotics.com/autonomous-weeder, last accessed on 20/7/2022.

[CR7] Dahm, J. (2022). *Germany fears seasonal labour shortages as Ukraine war rages on*. https://www.euractiv.com/section/agriculture-food/news/germany-fears-seasonal-labour-shortages-as-ukraine-war-rages-on/, last accessed on 20/7/2022.

[CR8] De Witte T (2019). Economic perspectives of small autonomous machines in arable farming. Journal für Kulturpflanzen.

[CR9] Ducksize (2022). *Farming robots to help you grow sugar beets*. https://www.ducksize.com/robots-for-beets, last accessed on 20/7/2022.

[CR10] Ecorobotix (2022). *AVO, our vision for the future: autonomous weeding (in development)*. https://ecorobotix.com/en/avo/, last accessed on 20/7/2022.

[CR11] European Commission (2022). Farm to Fork: New rules to reduce the risk and use of pesticides in the EU (2022). https://ec.europa.eu/commission/presscorner/detail/en/qanda_22_3694, last accessed on 20/7/2022.

[CR12] FarmDroid (2022). *The product sheet FD20: Automatic sowing and weeding of crops*. https://farmdroid.dk/en/product/, last accessed on 20/7/2022.

[CR13] Farmers Weekly (2021). *Solar-powered robot drills and weeds on Shropshire farm*. https://www.fwi.co.uk/machinery/technology/solar-powered-robot-drills-and-weeds-on-shropshire-farm, last accessed on 20/7/2022.

[CR14] Gallardo RK, Sauer J (2018). Adoption of labor-saving Technologies in Agriculture. Annual Review of Resource Economics.

[CR15] Heinrichs, J., Kuhn, T., Pahmeyer, C., & Britz, W. (2021). Economic effects of plot sizes and farm-plot distances in organic and conventional farming systems: A farm-level analysis for Germany. *Agricultural Systems*, 187. 10.1016/j.agsy.2020.102992.

[CR16] John Deere (2021). *John Deere launches See & Spray™ Select for 400 and 600 Series Sprayers*. https://www.deere.com/en/news/all-news/2021mar02-john-deere-launches-see-and-spray-select/, last accessed on 20/7/2022.

[CR17] Khanna M, Atallah SS, Kar S, Sharma B, Wu L, Yu C, Chowdhary G, Soman C, Guan K (2022). Digital transformation for a sustainable agriculture in the United States: Opportunities and challenges. Agricultural Economics.

[CR18] Kunz C, Weber J, Gerhards R (2015). Benefits of Precision Farming Technologies for mechanical weed control in soybean and Sugar Beet—Comparison of Precision Hoeing with Conventional Mechanical Weed Control. Agronomy.

[CR19] KTBL (Kuratorium für Technik und Bauwesen in der Landwirtschaft) (2022). *Betriebsplanung Landwirtschaft 2022/23 (In English: Agricultural operational planning 2022/23)* ISBN 978-3-945088-91-3. http://www.ktbl.de/shop/produktkatalog/19531

[CR20] KTBL (Kuratorium für Technik und Bauwesen in der Landwirtschaft) (2020). *Leistungs-Kostenrechnung Pflanzenbau (In English: Performance cost accounting of crop production)*. https://daten.ktbl.de/dslkrpflanze/postHv.html#Ergebnis, last accessed on 20/7/2022.

[CR21] KTBL (Kuratorium für Technik und Bauwesen in der Landwirtschaft) (2019). *Methodische Grundlagen der Datensammlung „Betriebsplanung Landwirtschaft“ (In English: Methodical bases of data collection in agricultural operational planning)*. https://www.ktbl.de/fileadmin/user_upload/Artikel/Datensammlung/Methodik.pdf, last accessed on 01/02/2023.

[CR22] Lowenberg-DeBoer, J., Franklin, K., Behrendt, K., & Godwin, R. (2021a). Economics of autonomous equipment for arable farms. *Precision Agriculture*, 1–15. 10.1007/s11119-021-09822-x. 10.1007/s11119-021-09822-x PMC8154546 34075304

[CR23] Lowenberg-DeBoer J, Huang IY, Grigoriadis V, Blackmore S (2020). Economics of robots and automation in field crop production. Precision Agriculture.

[CR24] Lowenberg-DeBoer J, Behrendt K, Ehlers MH, Dillon C, Gabriel A, Huang IY, Kumwenda I, Mark T, Meyer‐Aurich A, Milics G, Olagunju KO, Pedersen SM, Shockley J, Rose D (2021). Lessons to be learned in adoption of autonomous equipment for field crops. Applied Economic Perspectives and Policy.

[CR25] MacLaren, C., Storkey, J., Menegat, A., Metcalfe, H., & Dehnen-Schmutz, K. (2020). An ecological future for weed science to sustain crop production and the environment. A review. *Agronomy for Sustainable Development*, *40*(4), 10.1007/s13593-020-00631-6.

[CR26] Montanarella L, Panagos P (2021). The relevance of sustainable soil management within the european Green Deal. Land Use Policy.

[CR27] Naïo, T. (2022). *Dino - Weeding robot for large-scale vegetable crops*. https://www.naio-technologies.com/en/dino/, last accessed on 20/07/2022.

[CR28] Olabisi L, Wang R, Ligmann-Zielinska A (2015). Why don’t more farmers go Organic? Using a stakeholder-informed exploratory Agent-Based model to represent the Dynamics of Farming Practices in the Philippines. Land.

[CR29] Pedersen, S., Fountas, S., & Blackmore, S. (2008). Agricultural Robots - Applications and Economic Perspectives. In Y. Takahashi (Ed.), *Service Robot Applications* IntechOpen. 10.5772/6048

[CR30] Pedersen SM, Fountas S, Have H, Blackmore BS (2006). Agricultural robots—system analysis and economic feasibility. Precision Agriculture.

[CR31] Pérez-Ruíz M, Slaughter DC, Fathallah FA, Gliever CJ, Miller BJ (2014). Co-robotic intra-row weed control system. Biosystems Engineering.

[CR32] Rübcke von Veltheim F, Heise H (2020). The AgTech Startup Perspective to Farmers Ex Ante Acceptance process of Autonomous Field Robots. Sustainability.

[CR33] Rübcke von Veltheim F, Theuvsen L, Heise H (2022). German farmers’ intention to use autonomous field robots: A PLS-analysis. Precision Agriculture.

[CR34] Shockley, J., Dillon, C., Lowenberg-DeBoer, J., & Mark, T. (2021). How will regulation influence commercial viability of autonomous equipment in US production agriculture? *Applied Economic Perspectives and Policy* Advance online publication. 10.1002/aepp.13178

[CR35] Shockley JM, Dillon CR, Shearer SA (2019). An economic feasibility assessment of autonomous field machinery in grain crop production. Precision Agriculture.

[CR36] Sørensen CG, Madsen NA, Jacobsen BH (2005). Organic farming scenarios: Operational analysis and costs of implementing innovative Technologies. Biosystems Engineering.

[CR37] Stokstad E (2017). New crop pest takes Africa at lightning speed. Science (New York N Y).

[CR38] Spykman O, Gabriel A, Ptacek M, Gandorfer M (2021). Farmers’ perspectives on field crop robots – evidence from Bavaria, Germany. Computers and Electronics in Agriculture.

[CR39] Virtanen, P., Gommers, R., Oliphant, T. E., Haberland, M., Reddy, T., & Cournapeau, D., … SciPy 1.0 Contributors. (2020). SciPy 1.0: Fundamental Algorithms for Scientific Computing in Python.Nature Methods, *17*,261–272. 10.1038/s41592-019-0686-2 10.1038/s41592-019-0686-2 PMC7056644 32015543

[CR40] Williams, C., & Horodnic, A. (2018). *Tackling Undeclared Work in the Agricultural Sector: European Platform Undeclared Work* https://ec.europa.eu/social/BlobServlet?docId=20424&langId=en, last accessed on 20/07/2022.

