# Supplementary material for: How much can farmers pay for weeding robots? A Monte Carlo simulation study
Source: Precis Agric. 2023 Apr 5:1–26. Online ahead of print. doi: 10.1007/s11119-023-10015-x (PMC10075499; doi:10.1007/s11119-023-10015-x)
Supplement: Supplementary file 1 — Supplementary Material 1 [file 11119_2023_10015_MOESM1_ESM.docx]

February 08, 2023

Dear Editor,

We are very grateful for the constructive comments and positive feedback from you and the reviewers. Now we are glad to resubmit our manuscript to Precision Agriculture.

The major changes we have made include the following:

1. We rearranged the description of our dataset to provide more information about Table 1 and Table 2, as well as the connections between them.
2. We added discussion on the current robot prices on the market and commented on how a multipurpose robot would affect the weeding costs at the end of the conclusion.
3. We added the exemplary data of conventional farming in the appendix.
4. We added an acknowledgement section to clarify the contribution of the interviewees. All people listed in our acknowledgement have agreed to be on the list, including their company/institute names.

In addition, we have gone through the manuscript again and avoided the use of first person pronouns. All footnotes are removed.

Last but not least, we are not entirely certain how to interpret the comment of Reviewer 2 about adding a literature review in the introduction. From our perspective, the current introduction already includes the most relevant literature so far, and we have a separate section literature review to discuss the existing studies in detail. Adding a literature review in the introduction would make the introduction rather long and make it difficult to follow the main argumentation. Thus, we would appreciate it if we could hear your opinion on whether the corresponding changes should be made or not.

Thank you very much for your precious time! We are looking forward to hearing from you again.

Sincerely,

Linmei Shang, Christoph Pahmeyer, Thomas Heckelei, Sebastian Rasch, and Hugo Storm
